# How Two Brains Make One Synchronized Mind in the Inferior Frontal Cortex: fNIRS-Based Hyperscanning During Cooperative Singing

**DOI:** 10.3389/fpsyg.2015.01811

**Published:** 2015-11-26

**Authors:** Naoyuki Osaka, Takehiro Minamoto, Ken Yaoi, Miyuki Azuma, Yohko Minamoto Shimada, Mariko Osaka

**Affiliations:** ^1^Department of Psychology, Graduate School of Letters, Kyoto UniversityKyoto, Japan; ^2^Department of Psychology, Graduate School of Human Sciences, Osaka UniversitySuita, Japan; ^3^Center for Baby Science, Doshisha UniversityKyoto, Japan; ^4^Center for Information and Neural Networks, Osaka UniversitySuita, Japan

**Keywords:** hyperscanning, fNIRS, cooperation, singing, humming, inferior frontal cortex

## Abstract

One form of communication that is common in all cultures is people singing together. Singing together reflects an index of cognitive synchronization and cooperation of human brains. Little is known about the neural synchronization mechanism, however. Here, we examined how two brains make one synchronized behavior using cooperated singing/humming between two people and hyperscanning, a new brain scanning technique. Hyperscanning allowed us to observe dynamic cooperation between interacting participants. We used functional near-infrared spectroscopy (fNIRS) to simultaneously record the brain activity of two people while they cooperatively sang or hummed a song in face-to-face (FtF) or face-to-wall (FtW) conditions. By calculating the inter-brain wavelet transform coherence between two interacting brains, we found a significant increase in the neural synchronization of the left inferior frontal cortex (IFC) for cooperative singing or humming regardless of FtF or FtW compared with singing or humming alone. On the other hand, the right IFC showed an increase in neural synchronization for humming only, possibly due to more dependence on musical processing.

## Introduction

People's daily life experiences testify to the fact that through cooperation with others we can achieve goals that we could not reach otherwise. Studies seeking to identify the responsible brain mechanisms for cooperation have been unable to reveal details about the synchronization of the neural activations (Frith and Frith, 1999). Consequently, most investigations of social interactions have measured brain activities in only one person at a given time and not the dynamic interaction of two brains simultaneously.

Synchronization during social interactions has been reported using different neuroimaging techniques. For example, functional magnetic resonance imaging (fMRI) has been to observe two participants during a simple interaction game (Montague et al., 2002) or neuroeconomics (King-Casas et al., 2005), and electroencephalography (EEG) for social interactions (Astolfi et al., 2011), card game (Babiloni et al., 2006), instrument playing (Lindenberger et al., 2009), and cooperative prisoner's dilemma games (De Vico Fallani et al., 2010).

Singing together is a form of cooperation seen in all cultures and makes a suitable model to study the neural mechanisms of synchronization (Mithen, 2005). In animal studies, the vocalizations of monkeys often have a synchronized musical nature to them. This property is heard most dramatically in the rhythmic chattering of gelades, which are close cousins of baboon, and the “duet” singing of paired gibbons (Geissmann, 2002). Additionally, a pair of wrens showed cooperation through males and females rapidly alternating singing syllables (Fortune et al., 2011). Even insects like orthoptera have been observed to show activity akin to duet singing (Bailey, 2003). In humans, the neural synchronization of cooperative singing may have evolutionarily adapted to make a bond of affection in order to strongly bind groups of people (Dunber, 2010).

Singing together is also attributed to the adaptation of “flow” (Csikszentmihalyi, 1990). “Flow” can be defined as the mental state of operation in which people performing an activity are fully immersed in a feeling of energized focus. Musicians and choir experience flow, which allows them to make a harmonized song that could not be made with a single participant.

Using magnetoencephalography (MEG), Gunji et al. found distinct cortical rhythmic changes in response to singing and humming consistent with the motor control related to sound production (Gunji et al., 2007). In the alpha band, the oscillatory changes for singing were most pronounced in the right premotor and bilateral superior parietal areas. They also found a high frequency band in Broca's area when participants imagined they were singing.

Recently, online and simultaneous two-brain scanning of subjects engaged in interactive tasks has become possible. This new approach, hyperscanning, can be performed using several methods of different spatial and temporal resolution, including MEG, electroencephalography (EEG), functional magnetic resonance imaging (fMRI), and functional near-infrared spectroscopy (fNIRS), to examine how two brains dynamically interact to make a synchronized mind. fNIRS indirectly estimates a brain's neuronal activity by measuring concentration variations in oxygenated hemoglobin (oxy-Hb) and deoxygenated hemoglobin (deoxy-Hb), which have different absorption spectra in the surface brain's blood flow during the task performed.

Only few studies have reported the brain dynamics of social interactions using fNIRS to measure two brains simultaneously. Jiang et al. (2012) found a significant increase in neural synchronization in the left inferior frontal cortex (IFC) during face-to-face dialog between partners but not during a non-face-to-face dialog, while Cui et al. (2012) found synchronization in the right superior frontal cortex during cooperative but not competitive video games. Interestingly, language-based cooperative dialog and video-based spatial cooperative games activated the left and right frontal cortex, respectively. The dialogs in Jiang et al. (2012) are likely related to verbal activity in Broca's area, which is in the left IFC, while the visuo-spatial cooperative tasks in Cui et al. (2012) are likely related to activity in the right middle to superior frontal cortex.

These distinct areas should be relevant to synchronized singing and humming (the act of singing with open- and closed-lips, respectively), since the production of words during singing should engage Broca's area in the left IFC, while the production of melody during humming would be more related to the right IFC and superior frontal cortex, as reported using dichotic listening (Kimura, 1964). To test this theory, we applied fNIRS to investigate the neural synchronization of two cooperative partners when singing and humming. We also examined the neuronal differences between single and pair (cooperatively) synchronized singing and humming under face-to-face and non-face-to-face conditions by simultaneously measuring two brains.

## Materials and methods

### Participants

Thirty adults participated in the singing experiment (15 pairs, mean age of 22 years; eight male pairs and seven female pairs), and twenty-eight adults (14 pairs, mean age of 21 years: nine male pairs and five female pairs) in the humming experiment. The gender of the participant-pairs was controlled with matched age, and participants in each pair were mutually unfamiliar and assumed independent pairs. We obtained written informed consent from all participants, and the experimental protocol was approved by the Osaka University Institutional Review Board. Participants were paid (5000 yen each) for their participation.

### Stimulus

Three popular Japanese nursery rhymes were selected for the experiments: Under the Spreading Chestnut Tree, School of Killifish, and Sunset with the Evening Glow, since all participants would be familiar with these songs. Participants were instructed to sing or hum a melody part of the song, which lasted for 20–30 s.

### Procedure

**Figure 1:**
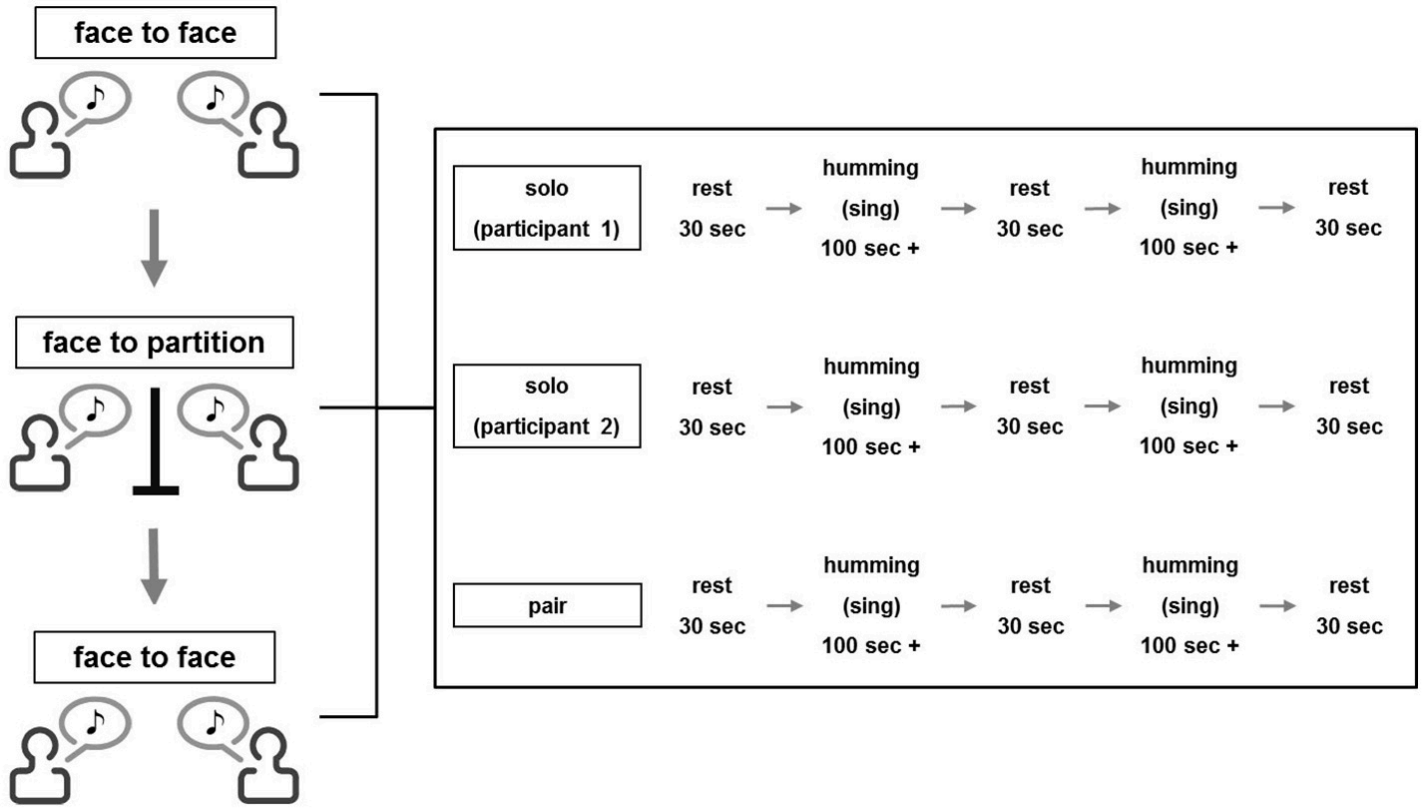
**Flow diagram of the task performances**.

In the singing experiment, participants were instructed to sing a song alone, listen to the partner's singing or sing with the partner. The order was counterbalanced across pairs. A time-course of each session is illustrated in Figure 1. In accordance with a previous fNIRS study (Cui et al., 2012), a 30-s rest period was given at the beginning of a session. Following the rest, participants sang a song alone or together or listened to the partner sing for about 100 s. When signing, the participant repeated the melody of one of the three songs described above (about 4–5 times) until the 100 s had passed and thereafter stopped singing. When listening, the participant was instructed to actively listen to the partner's singing and gaze at the partner's face. Then, another 30-s rest was given, which was followed by the second 100-s singing/listening interval. One more 30-s break was added at the end of each session. An experimenter measured the time with a stopwatch and instructed the beginning and end of each stage.

Participants performed three experimental sets, where each set consisted of the three conditions (single singing, cooperative singing, and listening). In the first set, two participants faced each other and performed the set while gazing at the partner's face (first face-to-face condition; FtF). In the second set, an opaque partition was placed between the participants (face-to-wall condition; FtW). In the third set, the partition was removed (second FtF condition). The same song was sung in each set, and the order of the songs was counterbalanced across pairs.

In the humming experiment, the procedure was identical to the singing experiment except that participants hummed the songs.

As the present experiment employed a three (participant 1 singing/humming, participant 2 singing/humming, and both participants singing/humming) × 3 (first FtF, FtW, and second FtF) within-subject design, a total of nine values for coherence increase was obtained in each pair.

### fNIRS data acquisition

**Figure 2:**
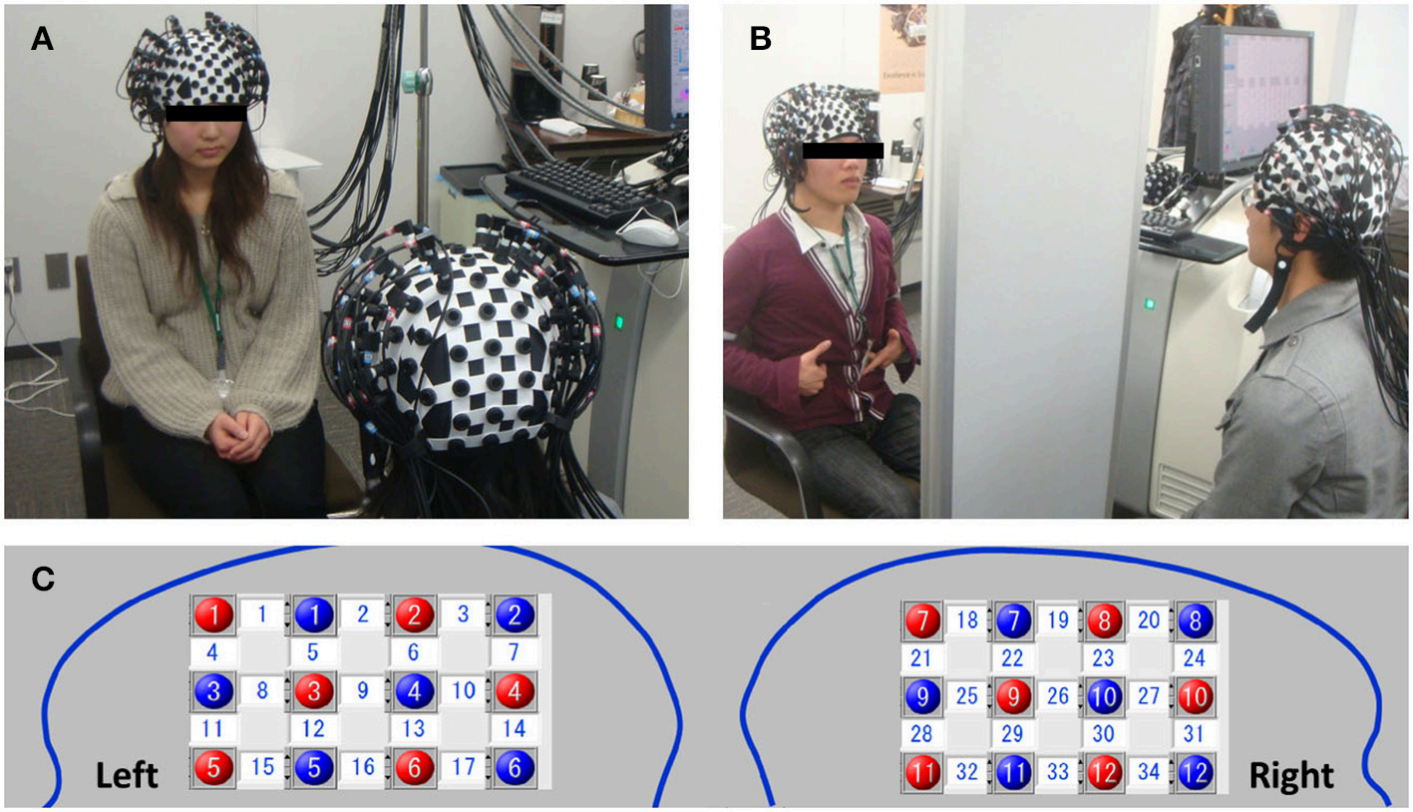
**A pair of participants sitting in face-to-face (FtF) (A) or face-to-wall (FtW) (B) conditions**. Red and blue indicate the positions of the emitters and detectors in the left and right head, respectively. Numbers in the white squares indicate the channels between the emitters and detectors **(C)**. These channels were connected to one fNRIS machine (LABNIRS). Channels 15 and 34 correspond to the left inferior and right IFC, respectively (note the nose position is reversed).

For the NIRS data acquisition, we employed a multichannel high-speed LABNIRS (Shimadzu, Japan) near-infrared spectroscopy measuring system to measure concentration variations in oxy-Hb and deoxy-Hb with easy operation. Combination of a three wavelength emitting system (780, 805, and 830 nm infra-red peak wavelengths) and a coupled photomultiplier detector tube achieved excellent sensitivity with scalability to increase the number of channels (up to 142 channels) according to the purpose and number of participants connected to a single machine. The absorption in these wavelength regions are caused mainly by oxy-Hb and deoxy-Hb, which have different absorption spectra, and the isosbestic point is in the vicinity of 805 nm. Therefore, if the molecular absorption coefficients of oxy-Hb and deoxy-Hb are known, the change in oxy-Hb and deoxy-Hb concentrations can be calculated by measuring the variation in absorption at two or more wavelengths. LABNIRS provides higher spatial resolution for high-density measurements and captures rapid cerebral blood flow signals in just 6 ms as compared with conventional sampling methods (http://www.shimadzu.com/an/lifescience/imaging/nirs/nirs3.html). A single 3 × 4 cm measurement patch was attached to a whole-head fiber holder (Flexible Adjustable Surface Holder; FLASH), which was placed on each participant's head so that the fronto-temporal cortex and neighboring parietal cortex activity could be measured (Figure 2). We selected L-shaped fibers for the measurement, and the patch was positioned symmetrically over each participant's right and left brain (Figure 2). For example, red 5 and blue 5 in the left brain (channel 15) indicate emitter and detector, respectively (Figure 2C). Bottom channels (15–17 in the left brain and 34–32 in the right brain were aligned to the Ca–Cp line (Talairach and Tournoux, 1988). Thus, in each patch, 12 emitters and 12 detectors were placed in the left and right brain of each participant, respectively, so that a total of 24 probes resulting in 34 measurement channels was employed for each participant. The sampling frequency we employed was 50 Hz.

### Data analysis

To analyze synchronization in the fNIRS data, we employed wavelet transform coherence (WTC) analysis to evaluate the relationships between the fNIRS signals generated by a pair of participants by calculating the cross-correlation as a function of frequency and time (Torrence and Compo, 1998). WTC shows the local correlation between two time series data (Cui et al., 2012). We used the wavelet coherence package (Grinsted et al., 2004) provided at the website: http://www.pol.ac.uk/home/research/waveletcoherence/. WTCs were performed in each channel across two participants, focusing on oxy-HB signals in accordance with Cui et al. (2012). For the analysis, we re-sampled oxy-HB time-series data to 10 Hz in each channel, simply averaging five consecutive data points.

### Random pair analysis

To exclude the possibility that the obtained coherence increase in cooperative singing/humming relative to single singing/humming was due to the two participants being engaged in the same task in the cooperative conditions but not in the single conditions, we performed a random pair analysis. The procedure was similar to that in Jiang et al. (2012), who tested coherence increase while two individuals were engaged in verbal communication. We selected two individuals from different pairs but sang the same song. Fifteen random pairs were made for the singing experiment and fourteen for the humming experiment. As the task duration differed across pairs, we adjusted the time-course data to be equal across the two individuals. That is, we specified the onset of singing/humming in each participant and defined the 30-s data before the onset as the pre-rest period and the 100-s data after the onset as the task period. We also specified the offset of singing/humming and defined the 30-s period after the offset as the middle- or post-rest period. WTC was applied to the two individual time-course data, and coherence increase was computed using the procedure described above.

For the random pair analysis, we determined the onset and offset of singing/humming and the rest period based on predetermined cue signals in the record. Therefore, the timing of singing/humming was matched between random pairs.

## Results

**Figure 3:**
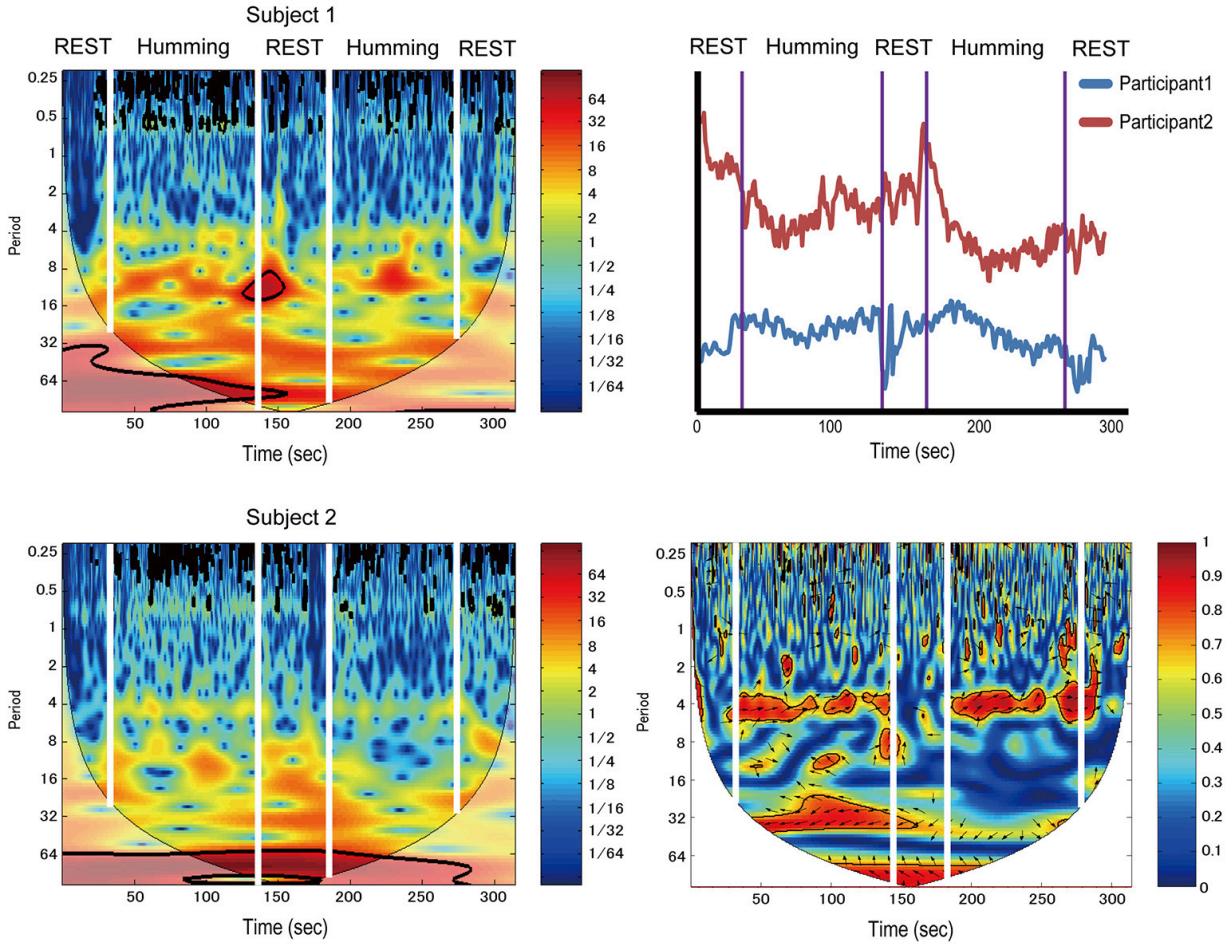
**Sample data from a pair of participants in the humming experiment**. The left two figures show continuous wavelet transform (CWT) data of different participants. The right-top figure shows time-course data of the same subjects. The right-bottom figure shows wavelet transform coherence (WTC). Color band indicates activation levels.

Data from a pair of participants in the humming experiment are shown in Figure 3. The left two figures show continuous wavelet transform (CWT) data of different participants. The right-top figure shows the time-course data from both participants. The right-bottom figure shows WTC. WTC between participants is meaningful if the CWT of each participant does not show change between the rest and task intervals, although our data shown in Figure 3 (left two figures) tended change a little at 4 s because the respiration changes CWT at 4 s.

**Figure 4:**
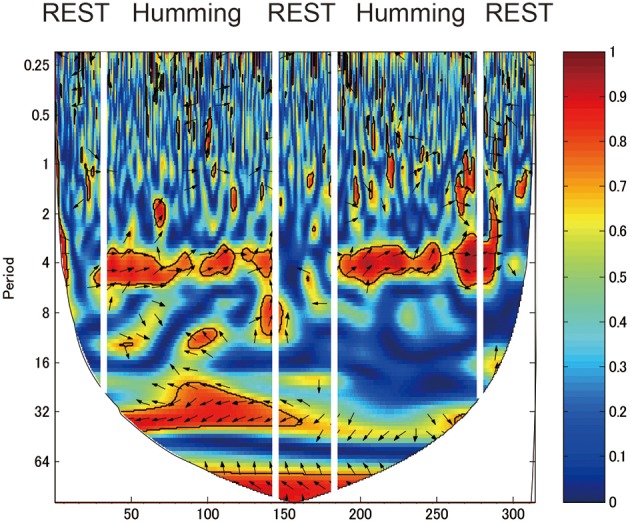
**Wavelet transform coherence (WTC) between the oxy-HB signal of two participants**. The panel shows WTC of data from the right channel 32. The red rectangles show frequency bands (period between 3.2 and 12.8 s), indicating when the task was performed.

**Figure 5:**
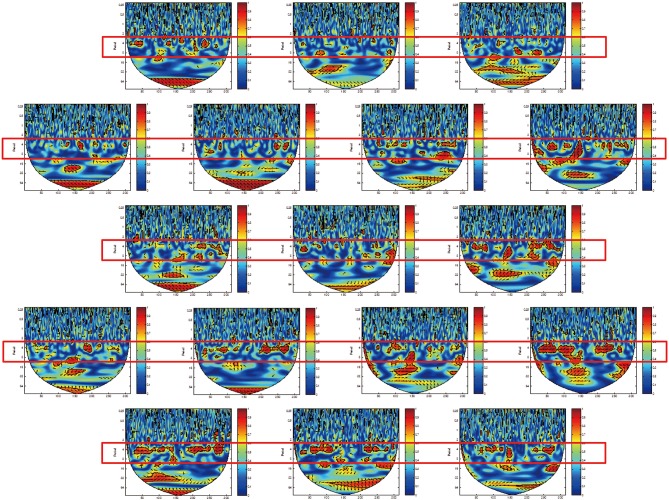
**Wavelet transform coherence (WTC) between the oxy-HB signal of two participants**. The panels show data from channels 18 (left top) to 34 (right bottom) in the right hemisphere. The red rectangles show frequency bands (period between 3.2 and 12.8 s), indicating when the task was performed.

We identified a frequency band that indicates the task was performed at approximately between 3.2 and 12.8 s (corresponding to a frequency of 0.3–0.08 Hz; Figures 4, 5, red rectangles). Cui et al. (2012) found a similar frequency band from data using a cooperative task in which the difference between the response times of both participants was smaller than a threshold time. Their frequency band includes the period of the trial (7 s), indicating that the coherence increase in their band is task-related. We assumed our cooperated singing/humming conditions were similar to their cooperative game. In our study, breathing of both participants played a critical role in synchronized singing, and singing occurs only when the breathing occurred at a specific period of about 4 s (frequency of respiration at about 15 breaths per min; Vaschillo et al., 2006). Right-, left-, and downward arrows indicates in-phase, out-of-phase, and direction of WTC between the raw oxy-HB signal of two participants, respectively (Figures 4, 5).

Coherence across two participants in the task phase (e.g., cooperative singing) was computed by averaging the coherence values of two singing blocks where two participants sang together for about 100 s. As Cui et al. (2012) suggested, we defined a coherence increase as the averaged coherence value in two task blocks minus the average coherence value in the rest block. That is, the averaged coherence value in the rest condition was subtracted from that of the singing/humming conditions, and the difference was used as an index of the neural synchronization increase between partners. Coherence increases were analyzed with a repeated ANOVA, including two factors with three levels in each. Because we repeated *F*-tests over 34 channels, *p*-values for the main effects and interactions were adjusted using the FDR method (*p* < 0.05).

### Coherence under the singing

In the cooperative pair, the coherence increase was greater in the cooperative singing condition in the left IFC and the right middle temporal cortex (Table 1) than in the single singing condition regardless of FtF or FtW. Inclusion of the opaque partition did not weaken the coherence in the cooperative singing. A repeated ANOVA showed a main effect of the type of singing in the left IFC (Ch 11 and 12) and the right middle temporal cortex (Ch 25) (*P* < 0.05, FDR corrected for multiple comparisons). The main effect was attributed to the greater coherence increase in the cooperative condition, as *post-hoc* multiple comparisons showed a significant increase in the cooperative condition compared with the single condition.

**Table 1:** **Brain regions activated under the cooperative condition as compared with the single condition**.

**Channel**	**Region**	**R/L**	***F*-values**	**η^2^*_*p*_***
**HUMMING EXPERIMENT**				
3	Parietal cortex	L	6.50	0.34
11	Inferior frontal cortex	L	5.24	0.29
12	Inferior frontal cortex	L	7.87	0.37
15	Inferior frontal cortex	L	11.02	0.46
17	Inferior temporal cortex	L	6.20	0.32
23	Middle frontal cortex	R	15.56	0.55
24	Middle frontal cortex	R	5.80	0.31
25	Middle temporal cortex	R	6.81	0.34
29	Middle temporal cortex	R	5.03	0.28
31	Inferior frontal cortex	R	6.10	0.32
32	Inferior temporal cortex	R	5.46	0.30
33	Inferior temporal cortex	R	7.13	0.35
34	Inferior frontal cortex	R	4.85	0.27
**SINGING EXPERIMENT**				
11	Inferior frontal cortex	L	11.55	0.45
12	Inferior frontal cortex	L	7.45	0.35
25	Middle temporal cortex	R	6.64	0.33

**Figure 6:**
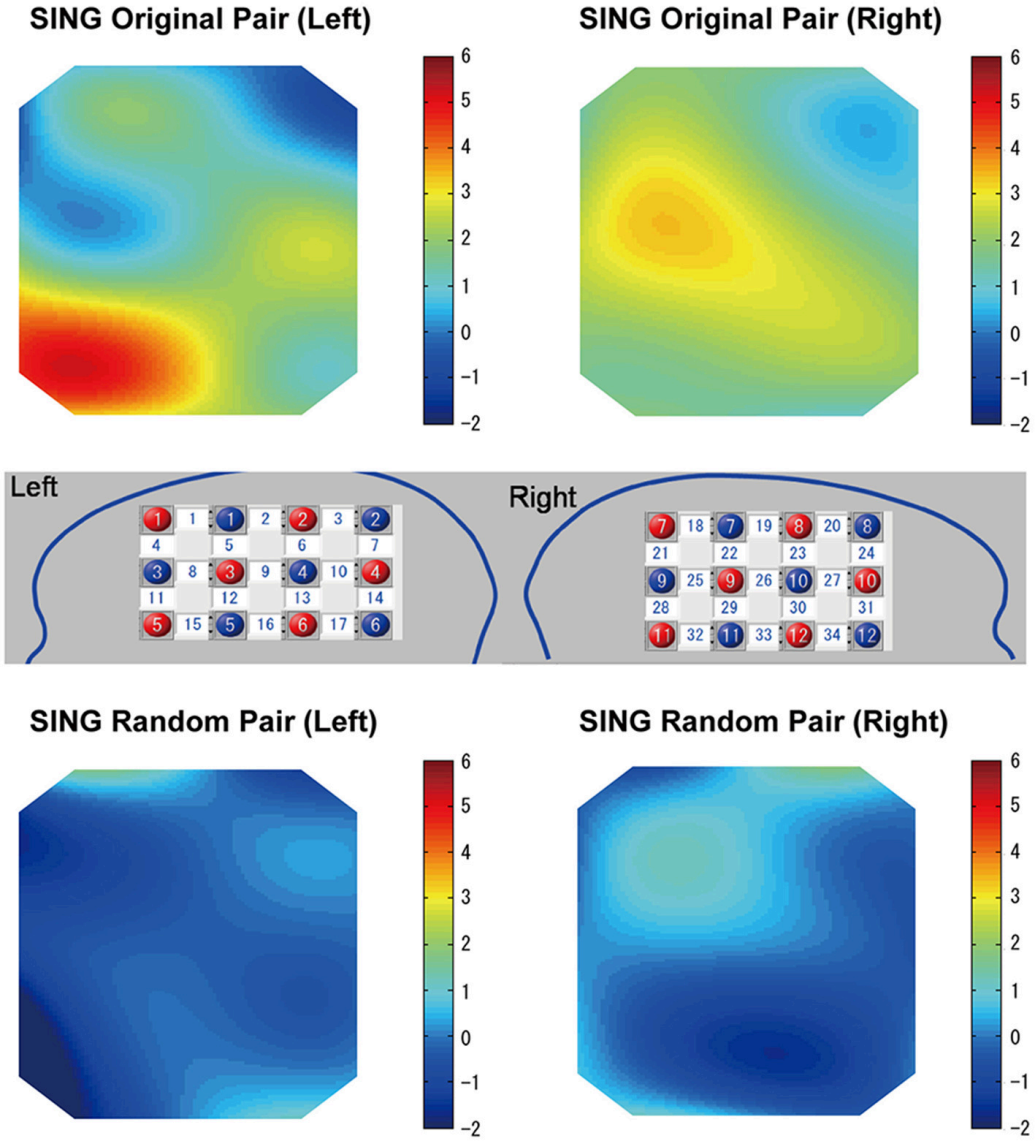
**Heat maps of WTC group data comparing cooperative and single singing for the subject pairs singing together (top)**. Heat maps of WTC group data comparing cooperative and single singing for randomly generated subject pairs **(bottom)**. Numbers in the white squares indicate the channels between the emitters and detectors.

Figure 6 (top) shows heat maps in which the coherence increase in the cooperative singing condition was compared with the single singing condition using a one-sample *t*-test for each channel. For the maps, we averaged the coherence increase in the cooperative condition and in the single condition (subject 1-sing and subject 2-sing) across the three visibility conditions (first FtF, FtW, and second FtF). Therefore, the heat maps correspond to T-maps smoothed by a spline correction method, which illustrates the channels that showed greater coherence increase in the cooperative condition than in the single condition. However, the coherence increase was equivalent across visibility (FtF vs. FtW) conditions (**Figure 8**). An ANOVA of data from the left IFC did not show a main effect of the visibility, *F*_(2, 28)_ = 0.80, *p* = 0.46, $\eta_{p}^{2}=0.05$. Similarly of the right IFC, a main effect of visibility was not significant, *F*_(2, 28)_ = 1.46, *p* = 0.25, $\eta_{p}^{2}=0.09$, either.

In the random pair, a coherence increase in the cooperative condition was not found (Figure 5, bottom). A repeated ANOVA did not show a main effect of singing type in all channels, nor a main effect of the visibility (*P* > 0.05). The interaction between factors was not significant in all channels (*P* > 0.05).

### Coherence under the humming

Similar to the singing experiment, in the cooperative pair, the coherence increase was greater in the cooperative humming condition than in the single humming condition, but in more brain areas, including the left parietal cortex, the bilateral IFC, the right middle frontal cortex, and the right middle temporal cortices (Table 1). Figure 4 shows WTC between the oxy-HB signal of two participants from channel 32 in the right hemispheres. Figure 5 shows the other WTC data from channels 18–34 in the right hemispheres. The coherence increase was independent of the visibility condition. A repeated ANOVA showed main effects of the type of humming in the bilateral IFC (Ch11, 12, 15, 34), the left middle frontal cortex (Ch23, 24), the right parietal cortex (Ch3), the right middle temporal cortex (Ch 25, 29) and the bilateral inferior temporal cortex (Ch17, 32, 33; *P* < 0.05. FDR corrected for multiple comparisons). Like the singing experiment, we made heat maps (Figure 6) that compared the coherence increase in the cooperative condition and in the single condition across the visibility conditions, finding a stronger coherence increase in the cooperative humming condition relative to the single condition. A main effect of the visibility condition was not significant in all channels (*P* > 0.05), and no interaction was obtained in all channels (*P* > 0.05).

In the humming experiment, however, the coherence increase in the left IFC was greater in the second FtF than in the FtW condition, but not in the right IFC (**Figure 8**). An ANOVA of the left IFC showed a significant main effect of the visibility, *F*_(2, 26)_ = 5.12, *p* = 0.01, $\eta_{p}^{2}=0.28$, and Sheffer's modified sequentially Bonferroni test showed a significant difference in coherence between the second FtF condition and FtW condition (*p* = 0.02). Such a main effect was not found in the right IFC, *F*_(2, 26)_ = 0.34, *p* = 0.73, $\eta_{p}^{2}=0.02$.

**Figure 7:**
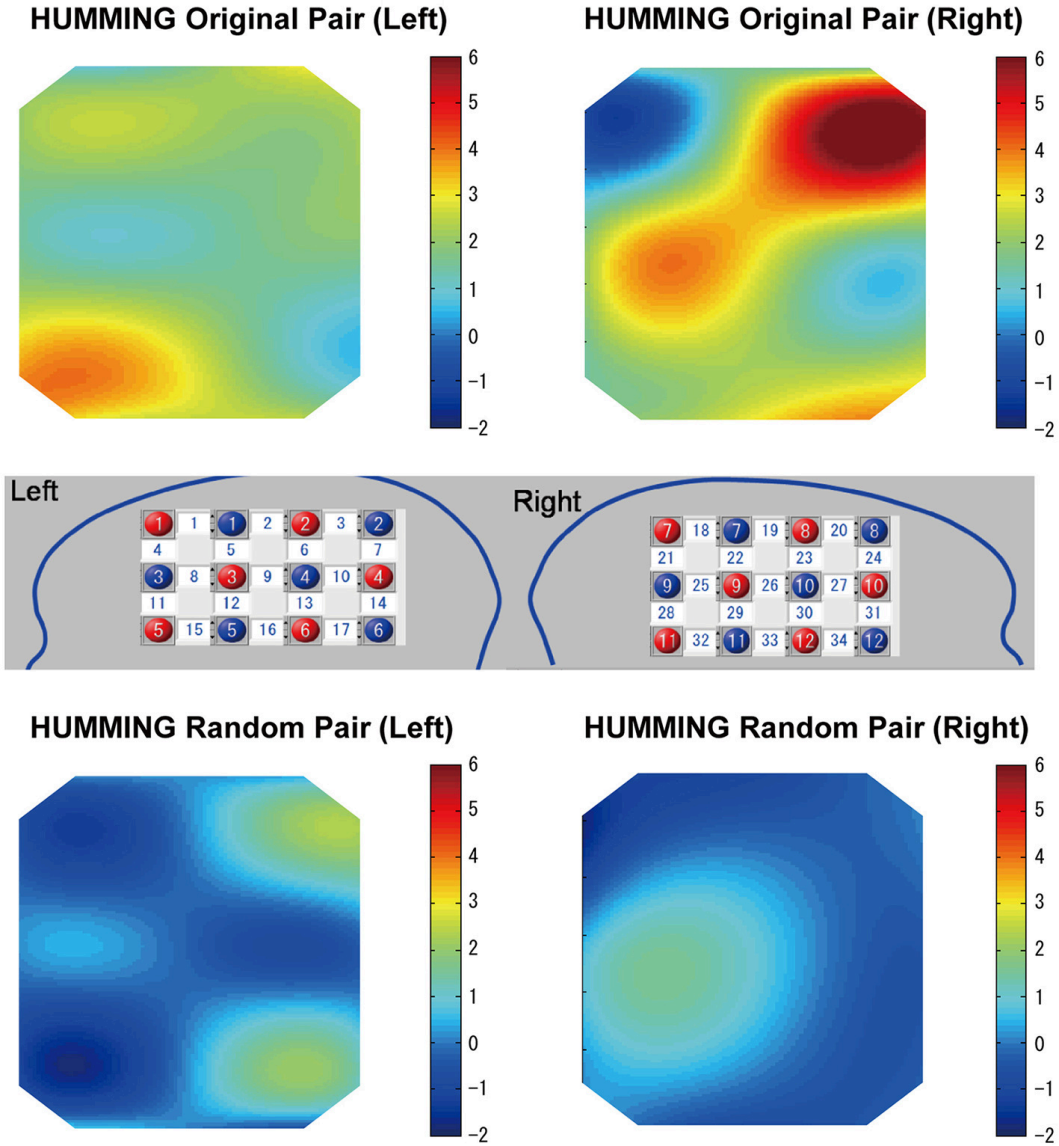
**Heat maps of cooperative (original) humming and single humming (group data)**. In the cooperative pair, stronger coherence was obtained in the bilateral inferior frontal cortices, the right middle frontal cortex and middle temporal cortex **(top)**. This result was not observed for the random pair **(bottom)**. Red and blue circles indicate the positions of the emitters and detectors in the left and right head, respectively. Numbers in the white squares indicate the channels between the emitters and detectors.

In the random pair, the coherence increase in the cooperative condition did not differ from that in the single condition (Figure 7). The repeated ANOVA neither showed main effects of humming type in all channels nor a main effect of the visibility conditions (*P* > 0.05). The interaction between factors was not significant in all channels (*P* > 0.05).

### Coherence under FtF and FtW

Because Jiang et al. (2012) found the left IFC (Ch 15) had stronger coherence in FtF, we examined this region and compared the coherence increase in cooperative singing/humming among the visibility conditions. In addition, the right IFC, which was in the equivalent position (Ch 34), was also analyzed. The coherence increase in the cooperative condition was compared among the visibility conditions. One-way repeated ANOVA was performed, and Sheffer's modified sequentially Bonferroni test was applied for multiple comparisons when a significant main effect was detected.

### Control experiment

In order to check whether our results were influenced by the task, we conducted an earphone control experiment in which the humming was presented through earphones (the sound was attenuated so that only the person with the earphones could hear the humming; *n* = 28; 14 pairs in the FtF condition in two sessions). This design could exclude the possibility that the synchronized activity was task-related. An ANOVA of this earphone experiment showed no main effect of the type of humming across all channels after applying false-discovery rate (FDR) correction (*p* < 0.05).

## Discussion

### Right and left IFC

In the present study, we employed a single fNIRS machine to measure the neural synchronization of two participants simultaneously while they were singing or humming together. We found, by applying inter-brain WTC analysis between two interacting brains, a significant increase in the neural synchronization in the left IFC. Interestingly, the left IFC is where Broca's area is located, which has been identified as key to singing (Brodmann Area BA44 and 45 of the dominant brain). Broca's area contributes to the utterance of the words of a song. Similar activation in Broca's region in the left IFC has been observed during face-to-face dialog between partners (Jiang et al., 2012). Along with the left IFC, the right IFC was activated during humming, which was attributed to a coordinated production of melody. Cui et al. (2012) found significant neural synchronization in the left IFC during cooperation but not during competition. The current study confirmed no WTC during single or random-paired conditions, which clearly indicates bilateral IFC activation in cooperative tasks. Therefore, both the left and right IFC are likely responsible for synchronizing two brains, with activation of the left IFC being superimposed for the bias of verbal expression. Along with the right IFC, the middle temporal cortex and middle frontal cortex are suggested to contribute to neural synchronization during humming. However, the activation of the superior frontal cortex reported by Cui et al. (2012) under the cooperation task was not observed, likely due to the difference in tasks.

Recent studies using hyperscanning to investigate the temporal and emotional aspects of music production have been reported (Lindenberger et al., 2009; Babiloni et al., 2011; Babiloni and Astolfi, 2014). Using EEG-based hyperscanning, Lindenberger et al. (2009) reported increased brain activity in the theta frequency band (4–7 Hz) of the prefrontal cortex during synchronous music production with the help of a metronome. Similarly Babiloni et al. (2012) simultaneously recorded the brain activities of saxophonists playing music in an ensemble and reported a correlation between empathy and alpha desynchronization in the right ventral-lateral frontal gyrus (BA 44/45). Our findings of activation in the right IFC under cooperative humming are in good agreement with these data. However, we found inter-brain oscillatory frequency bands of 0.3–0.08 Hz. The difference in frequencies can be explained in terms of the slow hemodynamic delay of about 3 s measured by fNIRS as compared with the fast waves measured by EEG.

### Face-to-face cooperation

**Figure 8:**
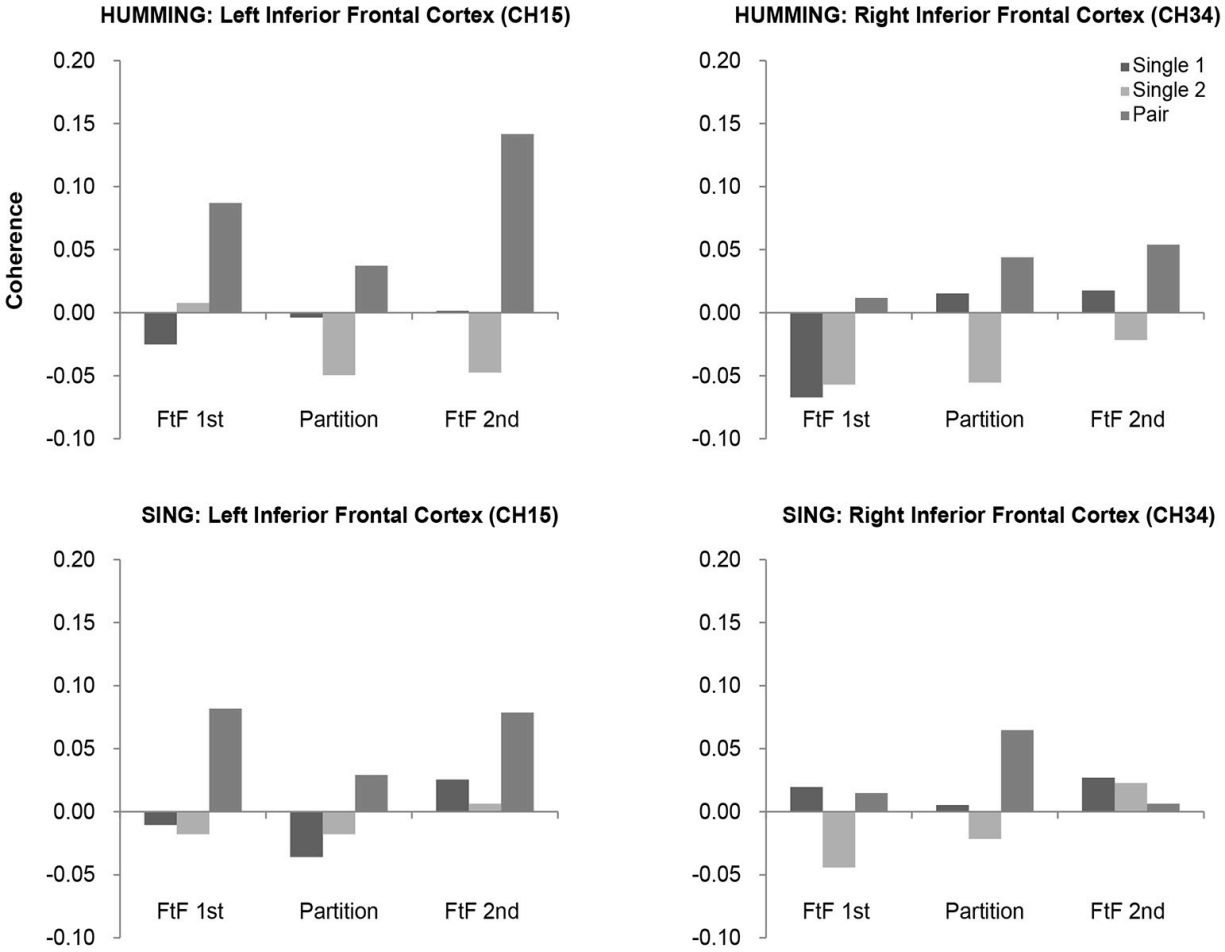
**Coherence values in the bilateral inferior frontal cortices (IFC) in the humming and singing experiments**. In the humming experiment, the bilateral IFC showed greater coherence in the cooperative condition than in the single condition across sessions (FtF and FtW). In the singing experiment, greater coherence in the cooperative condition was found only in the left IFC.

FtF social interactions are likely critical for synchronizing cooperation. Our study revealed FtF relatively tended to enhance activity of the left IFC under humming (Figure 8). FtW, however, showed negligible effects on the IFC and neighboring brain regions. These results are in good agreement with Jiang et al. (2012), who reported a significant increase in neural synchronization in the left IFC under FtF dialog, but not during back-to-back dialog, FtF monolog, or back-to-back monolog. In addition, we found that FtF played a critical role under humming, while FtW had negligible influence partly due to the importance of vocal rather than facial cooperation. As for why an increase in synchronization of the right IFC was seen for cooperative humming, only it could be that singing created a cognitive load.

A related study by Saito et al. (2010) that used fMRI reported pair-specific correlations of intrinsic brain activity during facial (eye) contact compared with non-paired subjects who were not in eye contact. They used an experimental paradigm in which the participants could recognize the gaze of the other on a screen on which there was also depicted other objects. Their results suggested that the right IFC was active in couples during conditions like FtF in our study.

### Social perspective

Cooperative singing may be beneficial to people whose sense of shared cooperation is weak. By singing together, an out-of-tune individual could be harmonized with an in-tune other, thus sharing joy through synchronized cooperation. Shared cooperation indicates the ability to create with others joint interactions and synchronized attention underlaid by cooperative motives (Tomasello, 2009). Furthermore, singing together enhances emotional relief and pleasure, and is expected to yield a sense of mutual trust and cooperation (Gaston, 1968; Anshel and Kipper, 1988).

Cooperative singing could also be partly interpreted as the result of mutual activations in the human mirror neuron system (MNS) of the prefrontal regions of two people. People have a tendency to imitate others using the MNS in order to conform to an indicator of group identity. Moreover, the MNS is likely located in the IFC and adjacent ventral premotor areas (Rizzolatti and Arbib, 1998; Iacoboni and Dapretto, 2006).

It is not surprising then that cooperative singing, which is a form of collective experience, gives rise to neural synchronization.

In summary, we examined how two brains make one synchronized mind using cooperative singing/humming between two people and hyperscanning. Hyperscanning allowed the observation of dynamic cooperation in which participants interacted with each other. We used fNIRS to record the brain activity of two brains while they cooperatively sang or hummed a song in FtF or FtW conditions. Inter-brain WTC between the two interacting brains showed a significant increase in the neural synchronization of the left IFC for both singing and humming regardless of FtF or FtW compared with singing or humming alone. On the other hand, the right IFC showed an increase in neural synchronization for humming only. Our data suggest, the application of hyperscanning during cooperative tasks could improve understanding of social cooperation.

### Conflict of interest statement

The authors declare that the research was conducted in the absence of any commercial or financial relationships that could be construed as a potential conflict of interest.
